# Modulation of Cytokine Expression in Human Myeloid Dendritic Cells by Environmental Endocrine-Disrupting Chemicals Involves Epigenetic Regulation

**DOI:** 10.1289/ehp.0901011

**Published:** 2009-08-28

**Authors:** Chih-Hsing Hung, San-Nan Yang, Po-Lin Kuo, Yu-Te Chu, Hui-Wen Chang, Wan-Ju Wei, Shau-Ku Huang, Yuh-Jyh Jong

**Affiliations:** 1 Department of Pediatrics and; 2 Graduate Institute of Medicine, College of Medicine, Kaohsiung Medical University, Kaohsiung, Taiwan;; 3 Department of Pediatrics, Kaohsiung Medical University Hospital, Kaohsiung Medical University, Kaohsiung, Taiwan;; 4 Center of Excellence for Environmental Medicine and; 5 Institute of Clinical Medicine, Kaohsiung Medical University, Kaohsiung, Taiwan;; 6 Johns Hopkins Asthma and Allergy Center, Johns Hopkins University School of Medicine, Johns Hopkins University, Baltimore, Maryland, USA

**Keywords:** dendritic cell, endocrine-disrupting chemical, epigenetic, mixed-lineage leukemia, MLL, nonylphenol

## Abstract

**Background:**

Exposure to environmental endocrine-disrupting chemicals (EDCs) is often associated with dysregulated immune homeostasis, but the mechanisms of action remain unclear.

**Objectives:**

The aim of this study was to test a hypothesis that EDCs regulate the functions of human dendritic cells, a front-line, immunoregulatory cell type in contact with the environment.

**Methods:**

We investigated circulating myeloid dendritic cells (mDCs) from five subjects and measured their responses, with or without coculture with autologous T cells, to two common EDCs, nonylphenol (NP) and 4-octylphenol (4-OP). EDC-associated cytokine responses, signaling events, and histone modifications were examined using ELISA, Western blotting, and chromatin immunoprecipitation (ChIP) assays, respectively.

**Results:**

In all cases, mDCs treated with NP or 4-OP demonstrated increased expression of tumor necrosis factor-α (TNF-α) but decreased baseline and lipopolysaccharide (LPS)-induced (interleukin) (IL)-10 production; the increase in TNF-α was partially reversible by an estrogen receptor (ER) antagonist. Activation of the MKK3/6-p38 signaling pathway marked the effect of NP on TNF-α expression, concomitant with enhanced levels of methyltranferase complex [mixed-lineage leukemia (MLL) and tryptophan-aspartic acid repeat domain 5 (WDR5)] in the nucleus and of trimethylated H3K4, acetylated H3, and H4 at the *TNFA* gene locus. Further, up-regulated TNF-α expression was significantly suppressed in NP-treated mDCs by a histone acetyltransferase inhibitor. In the presence of NP-treated mDCs, T cells showed increased levels of IL-13 but decreased expression of interferon-γ.

**Conclusions:**

These results suggest that NP and 4-OP may have functional effects on the response of mDCs via, in part, the ER, MKK3/6-p38 MAPK signaling pathway, and histone modifications, with subsequent influence on the T-cell cytokine responses.

In recent years, there has been increasing concern about exposure to ubiquitous environmental pollutants, including chemicals with endocrine-disrupting effects, and their impact on human health (Amaral Mendes 2002; Cheek et al. 1998; McLachlan 2001). This concern was highlighted by a recent survey of 1,455 adults revealing a potential link between exposure to environmental pollutants [e.g., bisphenol A (BPA)] and the occurrence of diseases (Lang et al. 2008). In the context of allergic diseases, including asthma, a recent meta-analysis of several epidemiologic studies in adult populations, mostly in occupational settings, showed associations between polyvinyl chloride (PVC) fume exposure and respiratory symptoms (Jaakkola and Knight 2008). Results from studies of children also revealed an association between PVC surface materials in the home and the risk of asthma and allergic rhinitis; notably, the concentration of diethylhexyl phthalate in indoor dust is associated with wheezing among preschool children (Bornehag et al. 2004; Kolarik et al. 2008). These findings may be relevant when the increase in the prevalence of allergic and autoimmune diseases in the industrialized countries over the last decades is taken into consideration (Asher et al. 2006).

Endocrine-disrupting chemicals (EDCs) are commonly found in the environment and originate from industrial and agricultural sources, including plant constituents, pesticides, and chemicals used in the plastics industry and in consumer products. Although the molecular mechanisms of EDC effects have not been defined, accumulated studies have revealed that EDCs can act via multiple mechanisms of action (Tabb and Blumberg 2006). Endocrine-disrupting effects are thought to be mediated, in part, by mimicking or blocking the action of the steroid hormones through both nuclear receptor–dependent and nonreceptor-dependent mechanisms, leading to differential target-gene transcription (Fisher 2004; Tabb and Blumberg 2006) and affecting not only the endocrine or reproductive systems but also the immune system (Ahmed 2000; Chalubinski and Kowalski 2006). Indeed, the results from *in vitro* and *in vivo* animal models have indicated that EDCs may act as immune modulators exerting their adjuvant effects at different levels of the immune regulatory network, including humoral immunity, cell survival, and cytokine synthesis (Chalubinski and Kowalski 2006). For example, nonylphenol (NP) and 4-octylphenol (4-OP), the breakdown products from domestic and industrial detergents, suppressed T-helper 1 (Th1) and enhanced Th2 cell development in a murine model (Kato et al. 2006). Environmentally relevant doses of tributyltin, another notable environmental pollutant, also promoted strong Th2 polarization and exacerbated antigen-induced airway inflammation in mice (Kato et al. 2004). These findings, together with the documented transgenerational effect of EDCs through epigenetic modifications (Anway et al. 2006), raise the possibility that EDCs such as NP and 4-OP may have significant impact on the genesis and the progression of host immunity.

Dendritic cells are primary and potent antigen-presenting cells with a critical role in initiation and progression of innate and adaptive immunity (Banchereau et al. 2000; Shortman and Liu 2002). Their strategic location throughout peripheral tissues and their role in surveillance for antigen exposure suggest that they are likely a target for EDCs; in fact, dendritic cells are able to extend dendrites into the lumen, presumably for sampling antigen (Rescigno et al. 2001). However, at present, it is unknown whether EDCs have any direct effect on human myeloid dendritic cells (mDCs). Considering the importance of mDCs in various disease contexts, we investigated the *in vitro* effects of two common EDCs, NP and 4-OP, on the generation of two regulatory cytokines, tumor necrosis factor-α (TNF-α) and interleukin (IL)-10, in human mDCs, as these two cytokines are important in pro- and anti-inflammatory processes, respectively (Asadullah et al. 2003; Mukhopadhyay et al 2006). The results herein provide evidence supporting the influence of EDCs on the function of mDCs, involving, in part, an estrogen receptor (ER)-dependent mechanism and activation of mitogen- activated protein kinase (MAPK), with subsequent modifications of histone structures associated with TNF-α expression in mDCs.

## Materials and Methods

### Isolation and analysis of mDCs

A total of five volunteer subjects were enrolled in this study, with a mean age of 33.2 years (range, 20–50 years). The study of human subjects was approved by the Institutional Review Board of Kaohsiung Medical University, and informed consent was obtained from each subject before blood samples were collected. Peripheral blood mononuclear cells (PBMCs) were isolated, and circulating mDCs were magnetically sorted using blood DC antigen (BDCA-1) cell isolation kits (Miltenyi Biotec, Bergisch Gladbach, Germany), following the manufacturer’s instructions. In all cases, the purity of isolated mDCs was > 90%. Purified mDCs (2 × 10^5^ cells/condition) were treated with 0.2 μg/mL lipopolysaccharide (LPS; from *Escherichia coli*, L0127:B8; Sigma, St. Louis, MO, USA), varying doses of NP or 4-OP, or a combination of LPS plus NP or OP and evaluated after various time points. Media alone was used as the control, and 17β-estradiol (E_2_; 10 μM, Sigma) was used in parallel cultures for comparison. To examine the involvement of the ER axis, we pretreated mDCs with an ER antagonist, ICI182,780 (10 μM; Sigma), 1 hr before treating the cells with NP or 4-OP. In some cases, after 1 hr treatment with 1 μM of one of three MAPK inhibitors (PD98059, SB203580, and SP600125; Sigma) and an IκB-α phosphorylation inhibitor, BAY11-7082, mDCs were stimulated with NP. To evaluate the effect of histone acetylation on TNF-α production, mDCs were treated with varying doses of anacardic acid (Calbiochem, San Diego, CA, USA), a histone acetyltransferase inhibitor. The production of cytokines in the culture supernatants was determined by ELISA (R&D Systems, Minneapolis, MN, USA) for IL-10 and TNF-α.

### Cytosolic and nuclear protein extraction

We treated 1 × 10^6^ mDCs with LPS (0.2 μg/mL), NP, or a combination of LPS plus NP for 1 hr and washed with ice-cold phosphate-buffered saline once and then resuspended. The cells were lysed in 10 mM HEPES, pH 7.9; 1.5 mM MgCl_2_; 10 mM KCl; 300 mM sucrose; 0.5% NP-40; and proteinase inhibitor cocktail (1.0 mM phenylmethanesulfonyl fluoride, 1.0 mM EDTA, 1 μM pepstatin A, 1 μM leupeptin, and 1 μM aprotinin) for 3 min on ice, then centrifuged at 6,500 rpm for 20 sec.

The supernatants were then collected for cytosolic protein analysis. The precipitants were resuspended using 20 mM HEPES, pH 7.9; 1.5 mM MgCl_2_; 420 mM NaCl; 1 mM dithiothreitol (DTT); 0.2 mM EDTA; 25% glycerol; and proteinase inhibitor cocktail on ice for 30 min, then centrifuged at 12,000 rpm for 5 min. The supernatants (nuclear lysate) were diluted with an equal amount of 20 mM HEPES, pH 7.9; 10 mM KCl; 1 mM DTT; 0.2 mM EDTA; 25% glycerol; and proteinase inhibitor cocktail.

### Western blotting

For Western blotting analysis, mDCs were treated with LPS (0.2 μg/mL), NP, or a combination of LPS plus NP for 1 hr and then lysed. Equal amounts of whole-cell lysates were analyzed by Western blotting with various antibodies, including anti-p65, antiphospho-p65 (pp65), anti-MKK-3/6, antiphospho-MKK3/6, anti-MAPKp38, and antiphospho-p38 antibodies (all from Santa Cruz Biotechnology, Santa Cruz, CA, USA). In some cases, nuclear extracts were obtained from 1 × 10^6^ mDCs stimulated with NP or 4-OP (10^−8^ M) for 1 hr and analyzed by Western blotting with anti–mixed-lineage leukemia (MLL) and anti-tryptophan-aspartic acid repeat domain 5 (WDR5) antibodies (Upstate Biotechnology, Waltham, MA, USA). Immunoreactive bands were visualized using horseradish peroxidase-conjugated secondary antibodies and the enhanced chemiluminescence (ECL) system (Amersham Pharmacia Biotech, Uppsala, Sweden).

### Stimulation of CD4^+^ T cells by mDCs and analysis of T-cell cytokines

We purified CD4^+^ T cells from PBMCs from the study subjects, using CD4 magnetic beads (Miltenyi Biotec) according to the manufacturer’s instructions. After treating the cells with medium alone or NP for 2 days, mDCs were cocultured with isolated CD4 T cells (1:10; a predetermined ratio for optimal cytokine analysis) for 5 days. Cell supernatants were collected for analysis of IL-13 and interferon-γ (IFN-γ) levels by ELISA assay.

### Chromatin immunoprecipitation assay (ChIP)

We performed ChIPs as described previously (Lee et al. 2003) with minor modifications: 5 × 10^5^ cells were treated with 1% formaldehyde for 10 min at room temperature, followed by sonication of DNAs and immunoprecipitation of chromatins overnight with antibodies for trimethylated H3K4 and acetylated histone H3 and H4 (Upstate Biotechnology, Temecula, CA, USA). Immune complexes were collected using a protein A slurry (Invitrogen, Carlsbad, CA, USA), and the DNA was reverse cross-linked, extracted, and quantified on a Taqman SDS 7900HT (Applied Biosystems, Foster City, CA, USA). For polymerase chain reaction (PCR) amplification of ChIP products, primers and probes were designed to analyze the proximal promoter and intronic enhancer regions of the *TNFA* gene as previously described (Sullivan et al. 2007), encompassing the following subregions relative to the transcription start site: TNF1 (+99 to −42); TNF2 (+32 to −119), TNF3 (−100 to −250), TNF4 (−195 to −345), and +1417, +720, and −1700. PCRs were run on the ABI 7700 Taqman thermocycler (Applied Biosystems). The relative intensities of the amplified products were normalized to the input DNAs as previously described (Lee et al. 2003; Sullivan et al. 2007).

### Statistical analysis

All data are presented as mean ± SD. Changes in cytokine levels were analyzed by using the Wilcoxon signed rank test. Differences between experimental and control groups were analyzed using the Mann–Whitney test. We consider a *p-*value < 0.05 to indicate significant between-group differences.

## Results

### NP and 4-OP modulate TNF-α and IL-10 levels in mDCs

**Figure 1:**
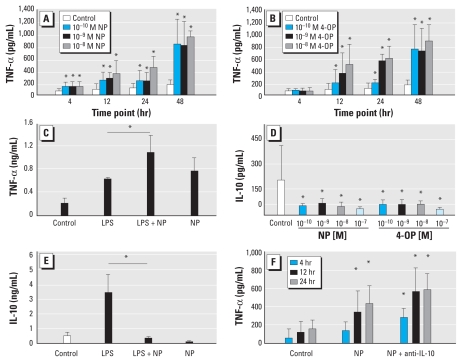
Effects of NP and 4-OP on production of TNF-α (*A,B,C,F*) and IL-10 (*D,E*) in mDCs evaluated at different time points after the initiation of the culture. (*A,B*) Levels of TNF-α after treatment with varied concentrations of NP (*A*) or 4-OP (*B*) at four time points. (*C,E*) Levels of TNF-α (*C*) and IL-10 (*E*) induced by 0.2 μg/mL LPS in mDCs in the presence or absence of 10^−8^ M NP at 48 hr. (*D*) Levels of IL-10 after treatment with NP or 4-OP for 48 hr. (*F*) Kinetic expression of TNF-α in mDCs treated with 10^−8^ M NP in the presence or absence of anti-IL-10 Ab. Data represent five independent experiments. *p < 0.05 compared with untreated cells in *A, B, D,* and *F*.

To examine the potential effect of NP and 4-OP on the expression of cytokines in mDCs, we treated mDCs with varying doses (10^−10^–10^−7^ M) of NP or 4-OP either alone or in combination with LPS (0.2 μg/mL) and evaluated cells at 4, 12, 24, and 48 hr after the initiation of the culture. The results showed that treatment of mDCs with either NP or 4-OP alone revealed significantly increased levels of TNF-α production, even at the lowest dose tested (10^−10^ M; Figure 1A,B). When LPS was added to the culture, there was a significant additive effect on the expression of TNF-α (Figure 1C). In contrast, NP and 4-OP appear to have an inhibitory effect on the expression of IL-10 (Figure 1D); although mDCs at resting stage without stimulation showed low but detectable levels of IL-10 expression, both NP and 4-OP inhibited the baseline level of IL-10. As expected, LPS induced a significant level of IL-10 production, but in the presence of NP, we found significantly reduced levels of LPS-induced IL-10 (Figure 1E). Moreover, the expression of TNF-α in mDCs treated with 10^−8^ M NP was further increased when neutralizing anti-IL-10 (0.1 μg/mL) antibodies were added to the culture, suggesting that NP-enhanced TNF-α expression appeared, in part, because of NP’s effect on the suppression of IL-10 expression (Figure 1F). During this culture period, there was no significant difference in the expression levels of DC maturation markers, including CD86, CD80, and HLA-DR, as judged by flow cytometry (data not shown).

### NP increased TNF-α production in mDCs partly via the ER

**Figure 2:**
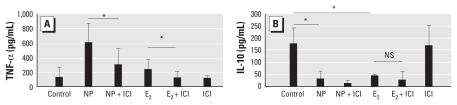
The regulatory activity of NP on cytokine expression is mediated, in part, via the ER. The levels of TNF-α (*A*) and IL-10 (*B*) in NP (10^−8^ M)-treated mDCs in the presence or absence of an ER antagonist, ICI182,780 (10 μM); 10 μM E_2_ was used in parallel cultures for comparison. NS, not significant. Data represent five independent experiments. *p < 0.05.

Because EDCs are able to serve as inflammatory inducers partly via the ER (Tabb and Blumberg 2006), we examined whether ER was involved in the regulatory activity of NP and 4-OP in mDCs. As shown in Figure 2A, the addition of an ER antagonist, ICI182,780, was able to partially suppress the levels of NP-induced TNF-α expression in mDCs. Moreover, mDCs treated with E_2_ showed increased levels of TNF-α production, and the addition of ICI182,780 receptor antagonist inhibited the induction of TNF-α expression (Figure 2A). These results suggested that NP-induced TNF-α expression in mDCs was, at least in part, mediated through the ER. However, ICI182,780 appeared to be incapable of blocking the suppressive effect of NP on IL-10 expression in mDCs, suggesting that NP-induced IL-10 expression may be an ER-independent pathway in mDCs (Figure 2B).

### NP increased TNF-α production in mDCs via the MKK 3/6-p38 MAPK pathway

**Figure 3:**
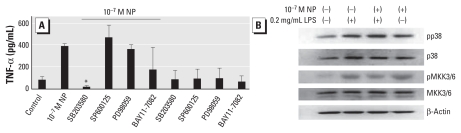
NP (10^−7^ M) increased TNF-α production in mDCs via the MKK 3/6-p38 MAPK pathway. (*A*) Levels of TNF-α expression in NP-treated mDCs in the presence or absence of 1 μM of a kinase inhibitor [SB203580 (p38-MAPK inhibitor), SP600125 (JNK inhibitor), or PD98059 (MEK1/ERK inhibitor)], or an IκB inhibitor (BAY11-7082). Data represent five independent experiments. (*B*) Western blot analyses of phosphorylated MAPKp38 (pp38), total p38, MKK3/6 (pMKK3/6), or MKK3/6; β-actin levels performed in parallel served as controls. *p < 0.05 compared with untreated cells.

Next, to investigate the NP-induced signaling events, we examined the effects of MAPK and NF-κB (nuclear factor kappa-B) inhibitors on NP-induced TNF-α expression in mDCs. Figure 3A shows that the addition of a p38-MAPK inhibitor (SB203580) suppressed NP-induced TNF-α expression, whereas other inhibitors—SP600125 (for JNK), PD98059 (for MEK1/ERK), and BAY11-7082 (for NFκB p65)—showed no significant inhibitory effect. Moreover, Western blot analyses of NP-treated or 4-OP–treated mDCs showed that, similar to the effect of LPS stimulation, EDC-treated mDCs demonstrated increased levels of phosphorylated p38 MAPK and MKK3/6 (Figure 3B); MKK3/6 serves as upstream signaling molecules of MAPKp38 (Hommes et al. 2003). No significant enhancement of phosphorylated NF-κB p65 was observed in NP-treated cells (data not shown). These results suggest the involvement of the MKK 3/6-p38 signaling pathway in NP’s effect on the induction of TNF-α expression.

### EDC-mediated histone modifications at the TNFA gene locus

**Figure 4:**
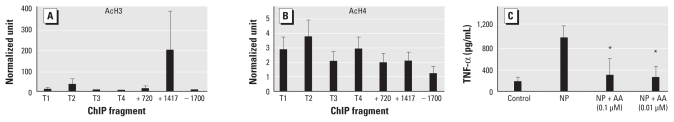
Involvement of histone acetylation in the regulatory effect of NP on TNF-α expression. ChIP analyses of the relative levels of acetylated H3 (AcH3; *A*) and acetylated H4 (AcH4; *B*) at the *TNFA* gene locus encompassing the following subregions relative to the transcription start site: TNF1 (T1; +99 to −42); TNF2 (T2; +32 to −119), TNF3 (T3; −100 to −250), TNF4 (T4; −195 to −345), +1417, +720, and −1700. The relative levels were normalized to the input DNAs and are shown as mean ± SD of five study subjects. (*C*) TNF-α expression levels in 10^−8^-M NP–treated mDCs in the presence or absence of varying doses of a histone acetyltransferase inhibitor, anacardic acid (AA). *p < 0.05 compared with NP treatment alone.

The expression of TNF-α is regulated in a complex network of molecular events, wherein epigenetic modifications occur both developmentally and in response to acute stimulation (Lee et al. 2003; Sullivan et al. 2007). In particular, recent evidence from analyses of monocytes and macrophages has shown that acetylated H3 and H4, together with trimethylated H3 lysine 4 (H3K4), mark active transcription in the *TNFA* gene locus (Sullivan et al. 2007). To examine whether the *TNFA* gene locus underwent histone modifications in mDCs as the result of the EDC treatment and MAPK activation, we performed ChIP analyses of mDCs treated with NP, using PCR primers corresponding to four overlapping subregions (−1700 and TNF1–4, covering the region between −345 and +99) in the TNFA promoter and two intronic regions (+720 and +1417) of the *TNFA* gene. Results showed that compared with those found in medium control cultures, significant histone modifications at the *TNFA* gene locus were present in NP-treated mDCs. As shown in Figure 4A, up-regulatedd TNF-α expression in NP-treated mDCs was accompanied by an increased level of histone 3 acetylation primarily in the intron sequence (+1417) of the *TNFA* gene, whereas increased histone 4 acetylation was associated predominantly with the proximal promoter regions of the *TNFA* gene (Figure 4B). In fact, the involvement of histone acetylation in the expression of TNF-α was further supported by the findings that the addition of a histone acetyltransferase inhibitor, anacardic acid, yielded significant suppression of TNF-α expression in NP-treated mDCs (Figure 4C).

**Figure 5:**
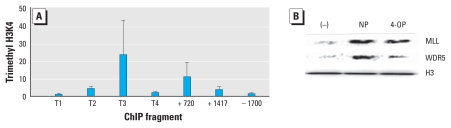
Effect of NP on H3K4 trimethylation at the *TNFA* gene locus and the expression of nuclear MLL and WDR5 in mDCs. (*A*) ChIP analyses of NP (10^−8^ M)-induced H3K4 trimethylation at the *TNFA* gene locus encompassing the subregions. (*B*) Western blot analyses of nuclear MLL and WDR5 levels in mDCs treated with NP (10^−8^ M) or 4-OP (10^−8^ M).

Moreover, ChIP analyses also revealed increased levels of trimethylated H3K4 at the proximal promoter subregion, TNF3, and an intronic region (+720) of the *TNFA* gene in NP-treated cells (Figure 5A). Trimethylation of H3K4 involves a sequential event from the initial recruitment of a methyltransferase complex, including a methyltransferase, MLL, and WDR5 protein scaffold, which in turn mediates dimethylation to trimethylation conversion of histone H3 at K4 (Dou et al. 2006). Therefore, we next evaluated whether NP and 4-OP could also influence nuclear MLL and WDR5 expression using Western blot analysis. Levels of MLL and WDR5 in the nuclear fractions of NP-treated mDCs were significant compared with those of medium controls (Figure 5B). These findings suggested, therefore, that the effects of NP and 4-OP are associated with their abilities in mediating differential histone modifications.

### NP-treated mDCs enhanced IL-13 production and suppressed IFN-γ production in T cells

**Figure 6:**
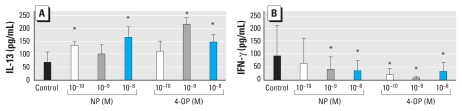
NP-treated mDCs enhanced IL-13 and suppressed IFN-α productions in T cells. Levels of IL-13 (*A*) and IFN-γ (*B*) of T cells after coculture with mDCs treated with varying doses of NP or 4-OP. Data represent five independent experiments *p < 0.05 compared with untreated cells.

We examined whether NP-treated mDCs influenced the cytokine responses in T cells. CD4^+^ T cells purified from PBMCs of the study subjects were cocultured with NP-treated or 4-OP–treated mDCs for 5 days. Cell supernatants were collected for IL-13 and IFN-γ measurement. IL-13 production in T cells was significantly increased after coculture with NP-treated and 4-OP–treated mDCs (Figure 6A), whereas IFN-γ production was suppressed in treated mDCs (Figure 6B). These data suggest that NP-treated or 4-OP–treated mDCs may affect the T-cell cytokine responses toward a Th2 phenotype after stimulation.

## Discussion

Dendritic cells have been recognized as a chief orchestrator of immune responses, and mDCs exert their regulatory functions, in part, through the release of cytokines after activation and/or inflammatory insults (Shortman and Liu 2002; Soumelis and Liu 2006). In the present study, we found that two common EDCs, NP and 4-OP, target and regulate cytokine responses of dendritic cells through mechanisms involving MAPK activation and histone modifications, suggesting the existence of an EDC–dendritic cell axis in the regulation of dendritic cell function and the subsequent T-cell responses. Results from recent epidemiologic and experimental studies have suggested the potential importance of exposure to EDCs in modifying the immune homeostatic state. Thus, understanding the targeting and regulatory pathways of EDCs, particularly in dendritic cells, would help in advancing the state of knowledge about EDC involvement in the development of immunologic diseases.

EDCs undergo significant bioaccumulation (Lalah et al. 2003), and various forms of metabolites can be detected in plasma and urine samples, the levels of which have served as a reference for estimating the levels of exposure. In a survey of subjects in New Zealand, Thomson et al. (2003) reported the plasma concentration of alkyl phenol of about 0.32 μg/L, which is between 10^−9^ M and 10^−8^ M. Also, in a study of 394 adults in the United States, Calafat et al. (2005) detected NP in 51% of samples, with levels ≥ 0.1 μg/L; the median and 95th percentile concentrations were < 0.1 μg/L and 1.57 μg/L, respectively. In the present study, the effective concentrations of NP and 4-OP for modulation of TNF-α expression were within the range of estimated exposure levels. The metabolism of NP is complex and yields a wide array of metabolites, thus decreasing the relative amounts of NP in the test urine samples. In fact, only 10% of ingested NP is excreted in the urine as NP or conjugated NP (Muller et al. 1998). Therefore, currently available data on exposure levels inferred from the measurement of NP in urine samples may represent only a small fraction of the NP that actually constitutes exposure. Moreover, given the known bioaccumulative effects of NP, the potential influence on dendritic cell function *in vivo* is likely to be greater.

In addition, our results showed that treatment of mDCs with NP or 4-OP resulted in preferential activation of the MKK 3/6-p38 MAPK pathway and differential effects on cytokine expression, associated with enhanced histone modifications at the *TNFA* gene locus. Also, NP and 4-OP inhibited the production of IL-10 but increased TNF-α production by mDCs, whereas ICI182,780, an ER antagonist, could reverse, at least in part, NP-induced TNF-α expression, suggesting the involvement of ER and MAPK activation in mediating NP’s effect. Further, the addition of a histone acetyltransferase inhibitor ablated the induction of TNF-α expression by NP. These results are consistent with the likelihood of ER-mediated chromatin remodeling in initiating gene transcription. However, ICI182,780 was unable to reverse the suppressive effect of NP and E_2_ on IL-10 expression in mDCs. Although the nature of this ER-independent mechanism is unclear at present, it has been shown that 4-OP has a functional effect on the activity of the aryl hydrocarbon receptor, an independent receptor (Bonefeld-Jørgensen et al. 2007). Further, NP is able to stimulate gene transcription mediated by the pregnane X receptor (PXR; a member of the nuclear receptor superfamily) (Masuyama et al. 2000), through its interaction with PXR, thereby facilitating the receptor-coactivator interaction. It is tempting to speculate that NP- (or 4-OP)-mediated TNF-α expression in mDCs may involve these two additional nuclear receptors. In addition, there is still a possibility that similar to other EDCs, NP and 4-OP may regulate hormonal responses by directly modulating cell-signaling pathways rather than interacting with hormone receptors (Tabb and Blumberg 2006). Cellular response to E_2_ may be mediated both by ER binding to the estrogen response element and by nonnuclear actions such as activation of signal-transducing pathways (ER independent) (Guo et al. 2006). It is particularly relevant that the suppressive effect of E_2_, NP, and 4-OP on the expression of IL-10 appears to be independent of the ER, suggesting the existence of alternative pathways. Moreover, our data showed that NP and 4-OP had a dose-independent effect on the expression of TNF-α. The reason is unclear at present, but this dose-independent phenomenon is not without precedents (Bevan et al. 2006; Bonefeld-Jørgensen et al. 2007), and suggests a rather complex mechanism operative in EDC’s effect on dendritic cells. Further detailed work is clearly needed to address these issues.

Although the detailed mechanisms and the extent to which the modifications occur upon exposure to EDCs remain unclear, the evidence provided here supports a role of EDCs in the regulation of mDC function through epigenetic modulation on the expression of cytokines, adding a new dimension to the existing regulatory network in mDCs. Epigenetic modification at the *TNFA* gene locus results from a concerted and complex network of regulation involving DNA methylation, histone modification, and chromatin remodeling. As has been noted in analysis of monocytes and macrophages (Sullivan et al. 2007), the present study showed that variable patterns of histone modifications were observed, and the primary regulatory regions associated with histone modifications could be identified in NP-treated mDCs. This variability may be due, in part, to the different levels of baseline and NP (or 4-OP)-induced TNF-α expression among the five subjects studied. Further study of additional subjects and detailed investigation with emphasis on defining the epigenetic mechanisms associated with EDC function would be important in defining the molecular basis of the involvement of EDCs in regulating immune response. An additional question that remains to be addressed is whether differential sensitivity of mDCs can be found between, for example, allergic versus nonallergic subjects, and our current studies would provide an experimental basis for addressing this possibility.

The use of plasticizers and the levels of their derivatives in the environment as EDCs have been increasing since World War II, and, incidentally, we have also witnessed an increase in prevalence of both autoimmune and allergic diseases (Asher et al. 2006). Although the exact cause for the increased prevalence is uncertain, the importance of environmental factors has been increasingly appreciated. One intriguing hypothesis has been the so-called hygiene hypothesis, which suggests that a lack of microbial exposure during critical periods in infancy increases the risk of allergic disease (Wills-Karp et al. 2001). Although not mutually exclusive, other hypotheses are equally plausible (Platts-Mills et al. 2005): The increased prevalence of allergic disease could be attributable to new immune adjuvant factors, for example, exposure to environmental pollutants such as diesel fuel exhaust particles and EDCs (Chalubinski and Kowalski 2006). The results of the present study would lend further support for this possibility. Furthermore, recent discoveries regarding aryl hydrocarbon receptor and environmental toxicant interaction and its influence on the balance of regulatory T and Th17 cells (Quintana et al. 2008; Veldhoen et al. 2008) highlight the potential importance of environmental exposure of various chemical pollutants and the development of immunologic diseases.

In summary, this study presents evidence supporting a role of NP and 4-OP in modulation of dendritic cell function through their ability to enhance TNF-α but suppress LPS-induced IL-10 expression. This effect involves the MKK3/6-p38 MAPK signaling pathway and histone modifications, leading to differential T-cell cytokine responses. TNF-α induces acute-phase response protein and plays an important role in the expression of inflammatory responses, whereas IL-10 is recognized primarily as an anti-inflammatory cytokine. Therefore, the functional effect of NP and 4-OP in inducing TNF-α, a proinflammatory cytokine, while decreasing IL-10 production in mDCs, suggests its proinflammatory function. Furthermore, NP-treated mDCs are able to polarize to a Th2 condition, suggesting a potential role of NP and 4-OP in the expression of allergic diseases. Although NP and 4-OP are common representative EDCs and their chemical structures are similar, the observed phenomena of NP and 4-OP may be results of the influence of specific categories of EDCs but not universal action of EDCs in general. Evaluation of additional EDCs is also important for further study.
